# Molecular Mechanism of Porcine Epidemic Diarrhea Virus Cell Tropism

**DOI:** 10.1128/mbio.03739-21

**Published:** 2022-03-14

**Authors:** Zhiwei Li, Zhiqian Ma, Linfang Dong, Ting Yang, Yang Li, Dian Jiao, Weiguo Han, Haixue Zheng, Shuqi Xiao

**Affiliations:** a State Key Laboratory of Veterinary Etiological Biology, Lanzhou Veterinary Research Institute, Chinese Academy of Agricultural Sciences, Lanzhou, China; b College of Veterinary Medicine, Northwest A&F University, Yangling, Shaanxi, China; c State Key Laboratory of Veterinary Etiological Biology, Lanzhou Veterinary Research Institute, Chinese Academy of Agricultural Sciences, Lanzhou, China; University of Calgary

**Keywords:** spike, cellular tropism, coronavirus, porcine epidemic diarrhea virus, reverse genetic analysis

## Abstract

In the 21st century, several human and swine coronaviruses (CoVs) have emerged suddenly and caused great damage to people's lives and property. The porcine epidemic diarrhea virus (PEDV), leading to enormous economic losses to the pork industry and remains a large challenge. PEDV showed extensive cell tropism, and we cannot ignore the potential risk of cross-species transmission. However, the mechanism of adaptation and cell tropism of PEDV remains largely unknown and *in vitro* isolation of PEDV remains a huge challenge, which seriously impedes the development of vaccines. In this study, we confirmed that the spike (S) protein determines the adaptability of PEDV to monkey Vero cells and LLC-PK1 porcine cells, and isolated exchange of S1 and S2 subunits of adaptive strains did not make PEDV adapt to cells. Further, we found that the cellular adaptability of rCH/SX/2016-S_HNXP_ depends on S1 and the first half of S2 (S3), and the 803L and 976H of the S2 subunit are critical for rCH/SX/2016-S1_HNXP_+S3_HNXP_ adaptation to Vero cells. These findings highlight the decisive role of PEDV S protein in cell tropism and the potential role of coronaviruses S protein in cross-species transmissibility. Besides, our work also provides some different insight into finding PEDV receptors and developing PEDV and other coronaviruses vaccines.

## INTRODUCTION

Coronaviruses are enveloped positive-sense and single-stranded RNA viruses (1). Several coronaviruses seriously threaten the health of both humans and domestic animals. An important reason why coronaviruses can persist and survive for a long time is that coronaviruses can easily spill across species (2). Human and other domesticated animals were susceptible to severe acute respiratory syndrome-coronavirus 2 (SARS-CoV-2) (3). Two porcine coronaviruses, swine acute diarrhea syndrome coronavirus (SADS-CoV) and porcine *deltacoronavirus* (PDCoV), might have a potential negative impact on the global economy and human health based on the observation of extensive cellular tropism *in vitro* (1, 4). Recently, PDCoV infection in humans has been reported (5).

Binding to the cognate receptor of the target species is a prerequisite for a virus to transmit to a new species, which for coronaviruses, is determined by the interaction between the viral S entry protein and its receptors (1, 6). The molecular basis of the broad host range of SARS-CoV-2 is that the SARS-CoV-2 spike binds to different species' angiotensin-converting enzyme 2 (ACE2) (7). PDCoV spike employs host aminopeptidase N (APN) as a strategy for transspecies transmission (1). PEDV is a causative agent of the porcine epidemic diarrhea, which shares similar clinical and pathological features with PDCoV and mainly causes severe watery diarrhea, vomiting, dehydration, and a high piglet mortality rate (8, 9). Similar to PDCoV, PEDV also showed extensive cell tropism (10–14). Therefore, theoretically, PEDV has the risk of cross-species transmission. The other data further support the evolutionary origin of PEDV from bats and potential cross-species transmission (15, 16). Hence, the study of the molecular basis of the host, tissue, or cellular tropism of PEDV is urgent.

The functional receptor of PEDV has not been identified, though it was discovered more than 40 years ago. APN was suggested as binding receptors for PEDV (10). Recent studies have shown that APN only contributes to PEDV infection, and APN-knockout pigs remained susceptible to PEDV infection (17, 18). Clathrin, caveolae, occludin, and transferrin receptor 1 participate in PEDV entry into cells (19–22). Unfortunately, these still cannot explain the molecular mechanism of PEDV cellular tropism and the reason why the PEDV isolation efficiency *in vitro* is extremely low, and the reason why the adaptive strain gradually loses its infectivity and partial immunogenicity during further passages in cell culture (23), which not only hinders PEDV basic research but also seriously hinders development process of PEDV vaccines. In addition, although PEDV is highly pathogenic to pigs, but not sensitive to some porcine-derived cell lines, which limits the study of PEDV pathogenesis and vaccines to a certain extent.

This study investigated the gene that determines PEDV adaptation to cells using three PEDV strains. CH/SX/2015 was isolated using Vero cells from the small intestine of PEDV-positive piglets in an outbreak farm, which belonged to the G1 group. PEDV CH/SX/2016 caused almost 100% mortality of piglets in another pig farm, which belonged to the G2 group, but repeated attempts to isolate the virus *in vitro* were unsuccessful. HNXP strain belongs to the G2 group and is susceptible to Vero and LLC-PK1 cells. Then, we constructed a series of chimeric recombinant viruses by exchanging the S, S1, or S2 genes. We confirmed that the cellular tropism of PED coronavirus depends on both S1 and S2 subunits, and the 803L and 976H of S2 subunits are critical for rCH/SX/2016-S1_HNXP_+S3_HNXP_ adaptation to Vero cells. This work highlights the key role of PEDV S protein in cell tropism and might inspire the identification of PEDV functional receptors and the development of vaccines of PEDV and other coronaviruses.

## RESULTS

### Virus Isolation.

**FIG 1:**
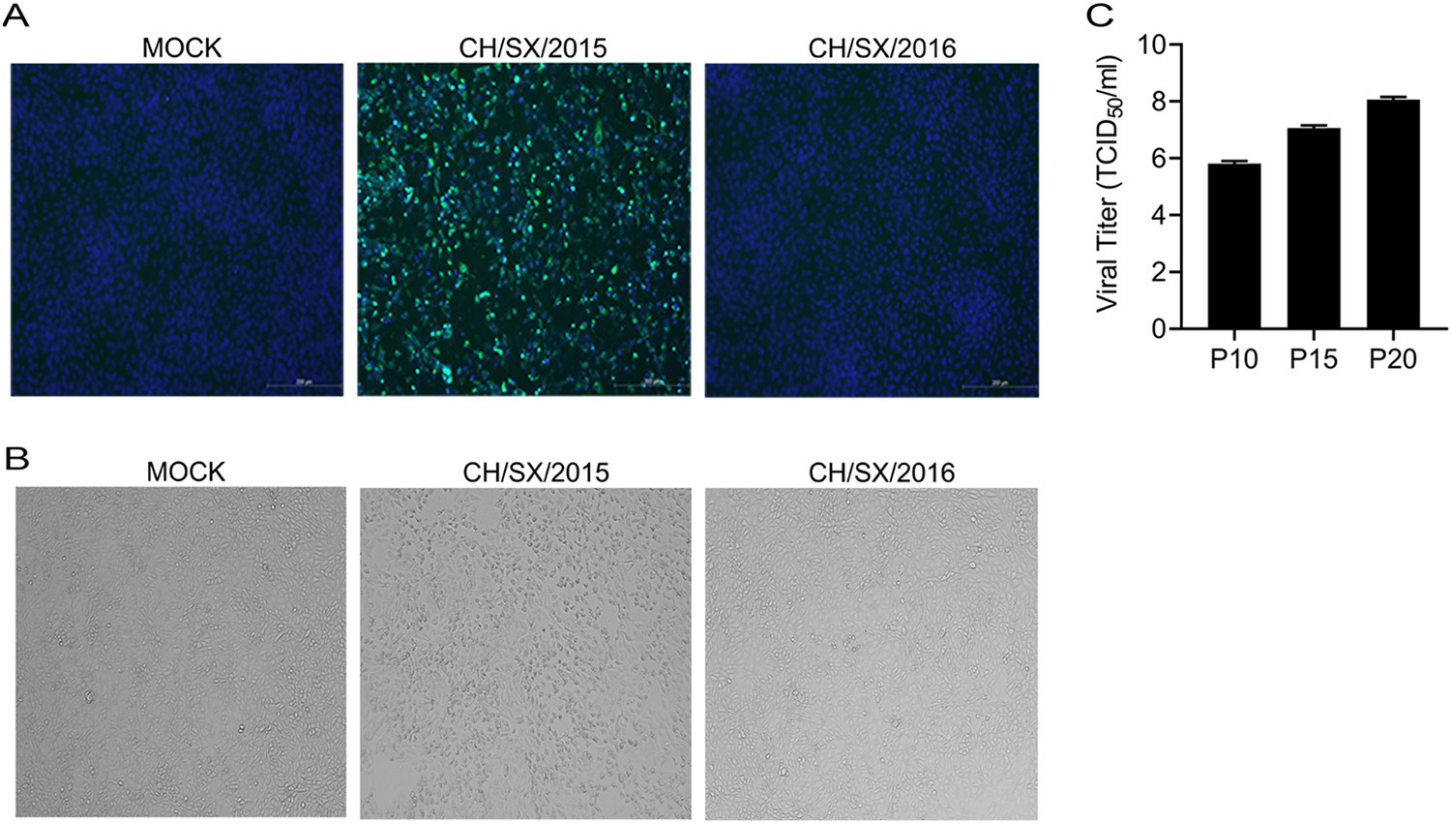
PEDV isolation. Vero cells were infected with PEDV. (A) Cells were fixed at 36 hpi, and immunofluorescence analysis was performed with anti-PEDV N (green) antibodies. (B) CPE was observed under a light microscope. (C) The viral titer was determined at 5-passage intervals.

Vero cells were used to isolate CH/SX/2015 and CH/SX/2016 from PEDV positive small intestinal tissues. A distinct cells' cytopathogenic effects (CPE) was observed in Vero cells inoculated with CH/SX/2015 small intestinal tissues after seven passages, but CH/SX/2016 was not, which were confirmed by immunofluorescence assay (IFA) using an anti-N polyclonal antibody (Fig. 1A). The CPE of CH/SX/2015 was characterized by cell shrinking and eventual cell detachment, no cell fusion, and syncytium formation (Fig. 1B). The PEDV CH/SX/2015 was further serially passed down a total of 20 passages, and the viral titer was determined at 5-passage intervals. The viral titer of the PEDV CH/SX/2015 reached 10^8^/mL at passage 20 (Fig. 1C).

### Construction and rescue of infectious PEDV rCH/SX/2015 and rCH/SX/2016 from the cDNA clone.

**FIG 2:**
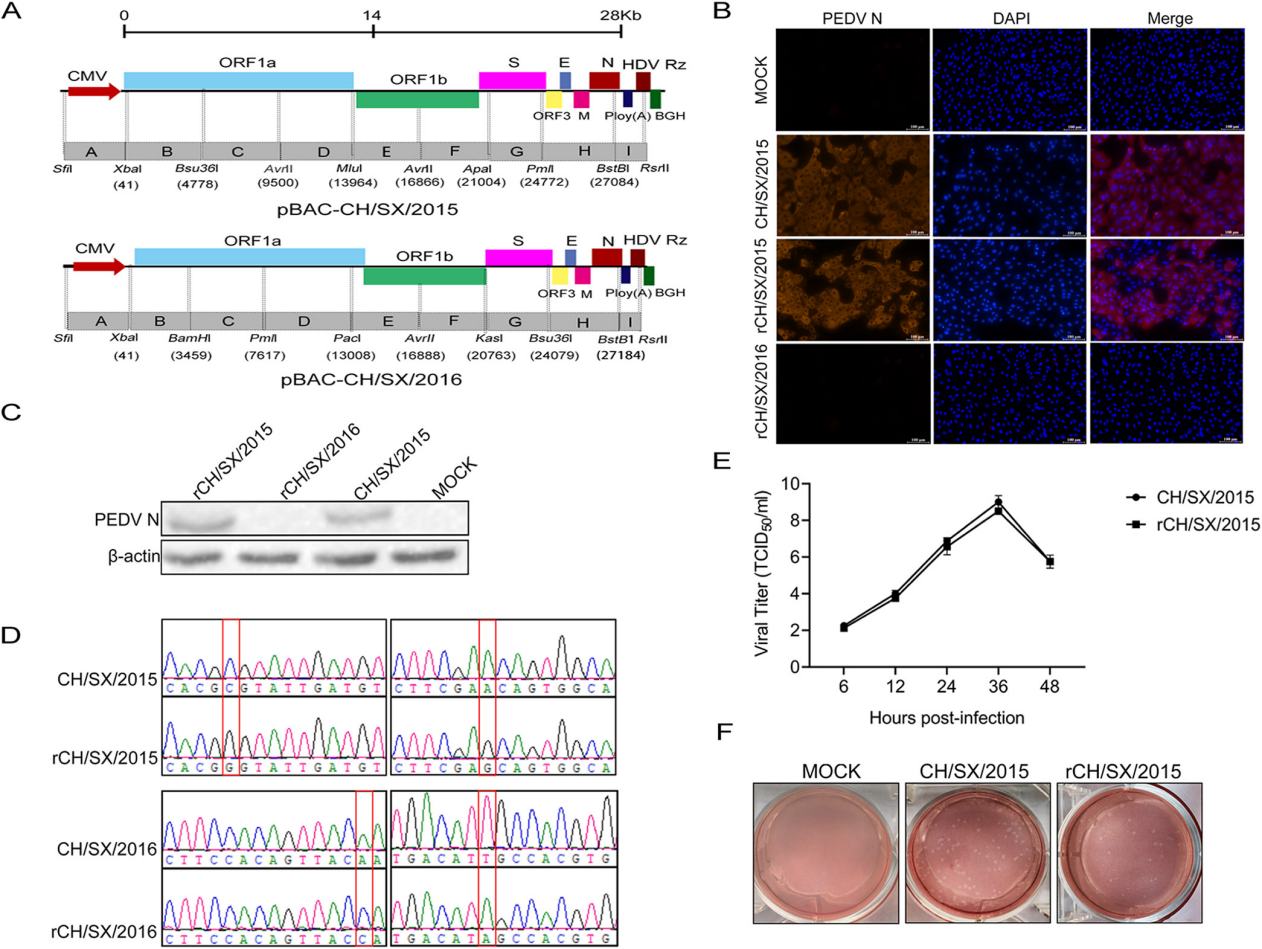
Construction and rescue of infectious PEDV rCH/SX/2015 and rCH/SX/2016 from the cDNA clone. (A) Schematic illustration of constructing rCH/SX/2015 and rCH/SX/2016. (B) Vero cells were infected with CH/SX/2015, rCH/SX/2015, and rCH/SX/2016. Infected cells were fixed at 36 hpi and immunolabeled with Rhodamine (TRITC) AffiniPure Goat Anti-Mouse IgG (H+L). Nuclei were labeled with DAPI (blue). (C) WB identified the rescue of rCH/SX/2015 and rCH/SX/2016. (D) The rCH/SX/2015 RNA of the three-passage (P3) virus stock was extracted and reverse transcribed. Then the marker mutation of rCH/SX/2015 was identified by sequencing. The recombinant plasmid rCH/SX/2016 was also identified by sequencing. (E) Vero cells in 12-well plates were infected with CH/SX/2015 and rCH/SX/2015. The supernatant was harvested at 6, 12, 24, 36, and 48 hpi and titrated on Vero cells. (F) Representative images of the plaque morphologies of CH/SX/2015 and rCH/SX/2015.

Each strain was divided into nine fragments, designated A-I to generate a construct spanning the entire genome of rCH/SX/2015 or rCH/SX/2016 (Fig. 2A). Then the fragments were connected into the pBeloBAC11 vector, which resulted in the generation of the rCH/SX/2015 or rCH/SX/2016 full-length infectious cDNA clone. The plasmids pBac-CH/SX/2015 and pBac-CH/SX/2016 were transfected into Vero cells at a confluence of about 80% in six-well plates, CPE was monitored daily. After 36 hpi, the CPE appeared in the cells transfected with pBac-CH/SX/2015, named rCH/SX/2015, but not in the cells transfected with pBac-CH/SX/2016, named rCH/SX/2016. The rCH/SX/2015 was identified further by IFA (Fig. 2B) and Western blot (Fig. 2C) using the mouse anti-N polyclonal antibody. Then sanger sequencing data revealed that the genetic markers, the cytosine (C) at the position 6786 was mutated to guanine (G), and the adenine (A) at position 8025 was mutated to guanine (G), respectively, which existed in the genome of rCH/SX/2015, but not in the genome of the parental CH/SX/2015 (Fig. 2D). The virus growth kinetics (Fig. 2E) and morphology of the plaques (Fig. 2F) showed that rCH/SX/2015 had the same growth characteristics as its parent strain CH/SX/2015. Thus, the recombinant PEDV rCH/SX/2015 was successfully rescued, but rCH/SX/2016 was not.

### The S gene is the key gene that determines PEDV adaptation to Vero cells and LLC-PK1 cells.

**FIG 3:**
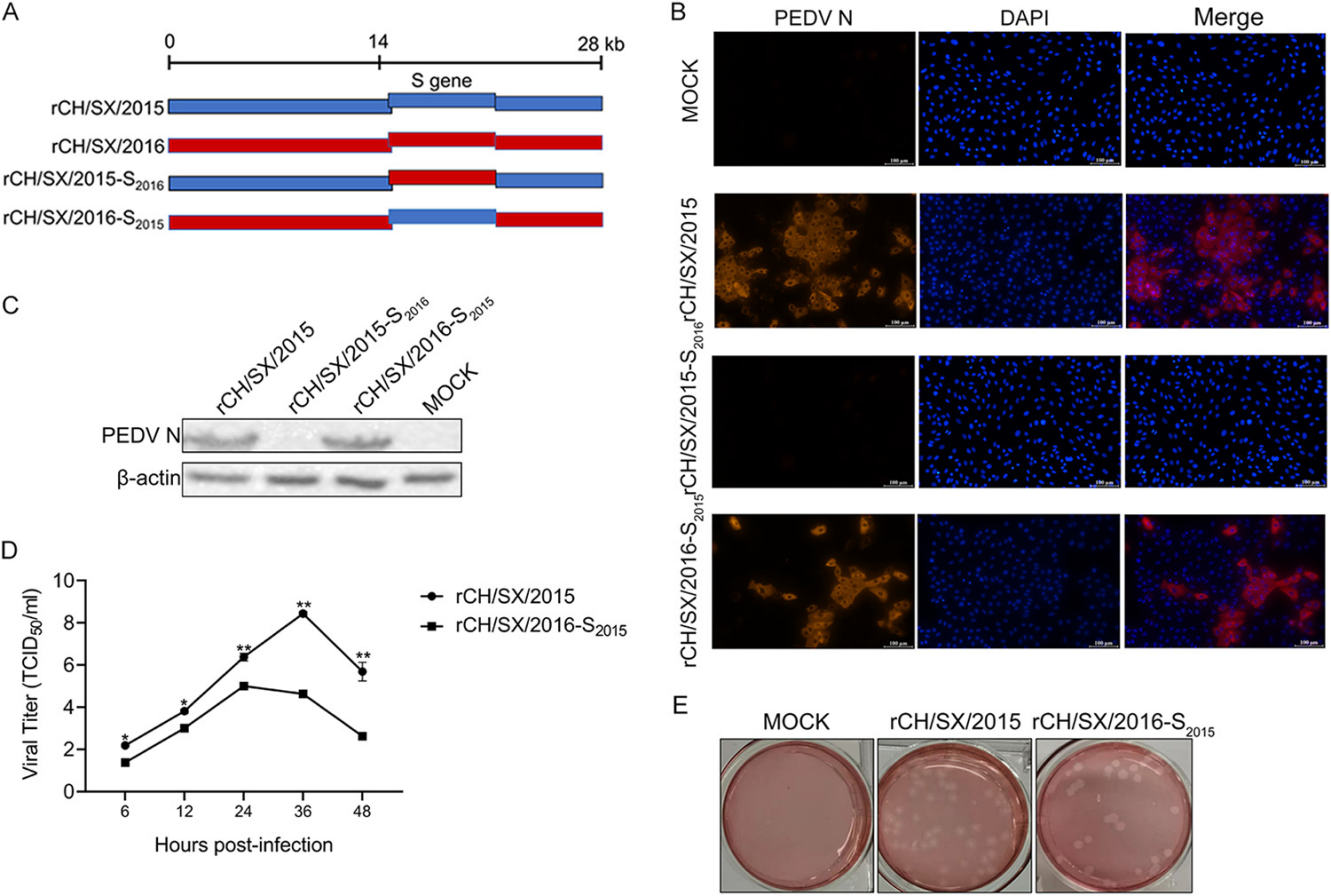
Adaptation of rCH/SX/2016-S_2015_ to Vero cells. (A) Schematic illustration of constructing rCH/SX/2015-S_2016_ and rCH/SX/2016-S_2015_. (B) Vero cells were infected with rCH/SX/2015, rCH/SX/2015-S_2016_, and rCH/SX/2016-S_2015_. Infected cells were fixed at 36 hpi and immunolabeled with Rhodamine (TRITC) AffiniPure Goat Anti-Mouse IgG (H+L). Nuclei were labeled with DAPI (blue). (C) WB identified the rescue of rCH/SX/2015, rCH/SX/2015-S_2016_, and rCH/SX/2016-S_2015_. (D) Vero cells in 12-well plates were infected with rCH/SX/2015 and rCH/SX/2016-S_2015_. The supernatant was harvested at 6, 12, 24, 36, and 48 hpi and titrated on Vero cells. (E) Representative images of the plaque morphologies of rCH/SX/2015 and rCH/SX/2016-S_2015_. Error bars indicate standard deviations. The level of significance was expressed as *, *P < *0.05, **, *P < *0.01 or ***, *P < *0.001.

According to the PEDV S protein function, we speculate that the S gene may be the key gene that determines PEDV adaptation to cells. To test this hypothesis, the S genes of rCH/SX/2015 strain adapted to Vero cells, and rCH/SX/2016 strain unadapted to Vero cells were exchanged (Fig. 3A). The rCH/SX/2016-S_2015_ strain was successfully rescued and identified by IFA (Fig. 3B) and Western blot (Fig. 3C), but rCH/SX/2015-S_2016_ was not successfully rescued (Fig. 3B and C). The growth kinetics showed that titers of recombinant PEDV rCH/SX/2016-S_2015_ were lower than rCH/SX/2015 (Fig. 3D). Nevertheless, the morphology of the plaques of rCH/SX/2016-S_2015_ was comparable to that of the rCH/SX/2015 virus (Fig. 3E).

**FIG 4:**
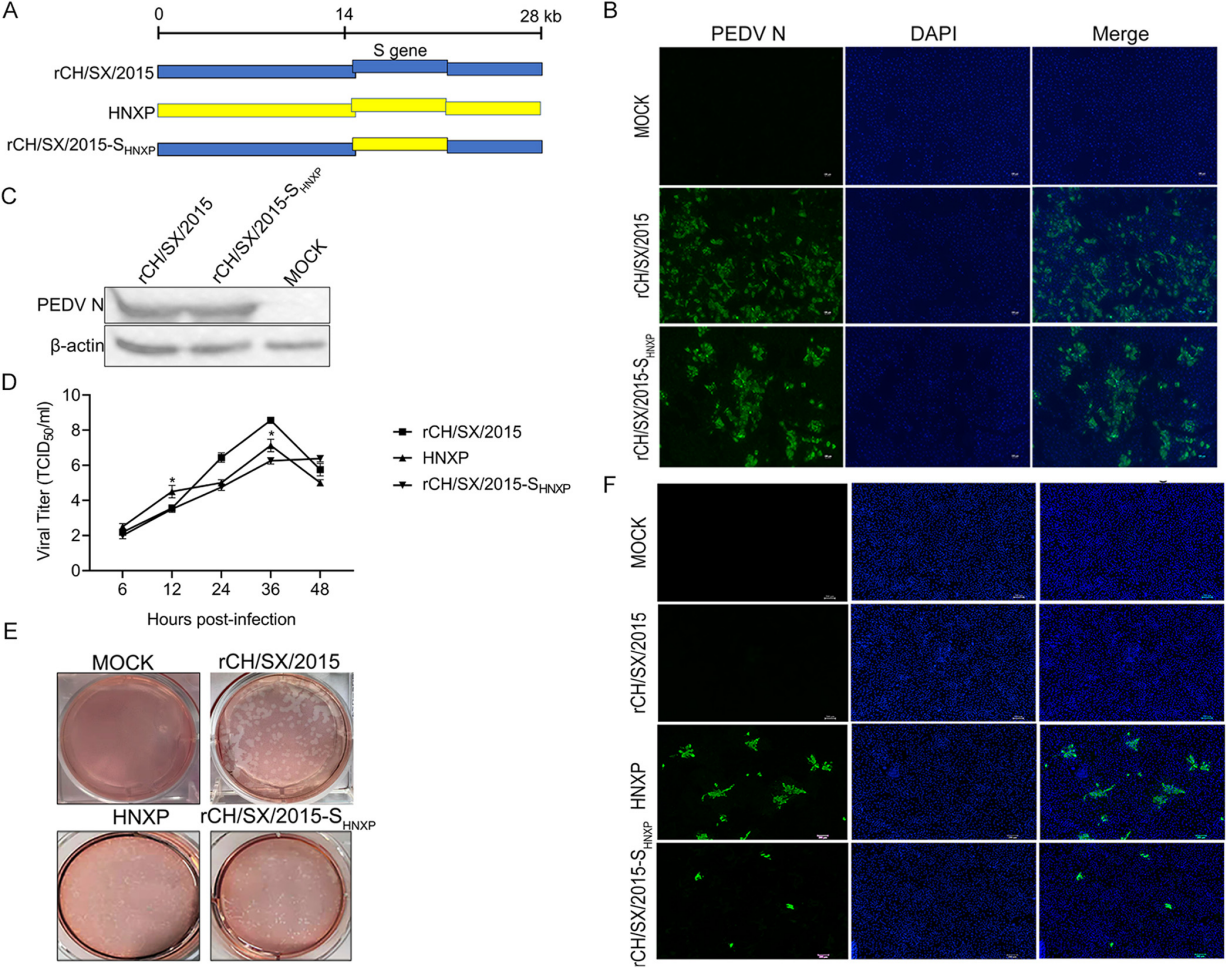
Adaptation of rCH/SX/2015-S_HNXP_ to Vero and LLC-PK1 cells. (A) Schematic illustration of constructing rCH/SX/2015-S_HNXP_. (B) Vero cells were infected with rCH/SX/2015 and rCH/SX/2015-S_HNXP_. Infected cells were fixed at 36 h postinfection and immunolabeled with Fluorescein (FITC)-AffiniPure Goat Anti-Mouse IgG (H+L). Nuclei were labeled with DAPI (blue). (C) WB identified the rescue of rCH/SX/2015 and rCH/SX/2015-S_HNXP_. (D) Vero cells in 12-well plates were infected with rCH/SX/2015, HNXP, and rCH/SX/2015-S_HNXP_. The supernatant was harvested at 6, 12, 24, 36, and 48 hpi and titrated on Vero cells. (E) Representative images of the plaque morphologies of mock, rCH/SX/2015, HNXP, and rCH/SX/2015-S_HNXP_. (F) LLC-PK1 cells were infected with rCH/SX/2015, HNXP, and rCH/SX/2015-S_HNXP_. Infected cells were fixed at 36 hpi and immunolabeled with Fluorescein (FITC)-AffiniPure Goat Anti-Mouse IgG (H+L). Nuclei were labeled with DAPI (blue). Error bars indicate standard deviations. The level of significance was expressed as *, *P < *0.05, **, *P < *0.01 or ***, *P < *0.001.

To further verify the role of the S gene in adapted porcine cells, we construct chimeric infectious cDNA clones rCH/SX/2015-S_HNXP_ (Fig. 4A). The S gene of rCH/SX/2015 strain unadapted to LLC-PK1 cells (Fig. 4F) was replaced with the S gene of HNXP strain adapted to Vero (Fig. 4D) and LLC-PK1 cells (Fig. 4F). The rCH/SX/2015-S_HNXP_ strain was successfully rescued and identified by IFA (Fig. 4B) and Western blot (Fig. 4C) in Vero cells. The replication of the recombinant virus in Vero cells was assessed by growth kinetics (Fig. 4D), the titers of rCH/SX/2015-S_HNXP_ virus were comparable to that of HNXP at 6 hpi and 24 hpi, but its titers were lower than HNXP virus at 12 hpi and 36 hpi. The morphology of the plaques of rCH/SX/2015-S_HNXP_ was comparable to that of the HNXP virus (Fig. 4E). LLC-PK1 cells were infected with rCH/SX/2015-S_HNXP_, and then rCH/SX/2015-S_HNXP_ was confirmed to infect LLC-PK1 cells, but the infection amount was significantly lower than that of parent strain HNXP by IFA (Fig. 4F). All the above results indicate that the S gene of PEDV is the key gene that determines the adaptability of PEDV to Vero cells and LLC-PK1 cells.

### The cellular adaptability of PEDV depends on the cooperation between S1 and S2.

**FIG 5:**
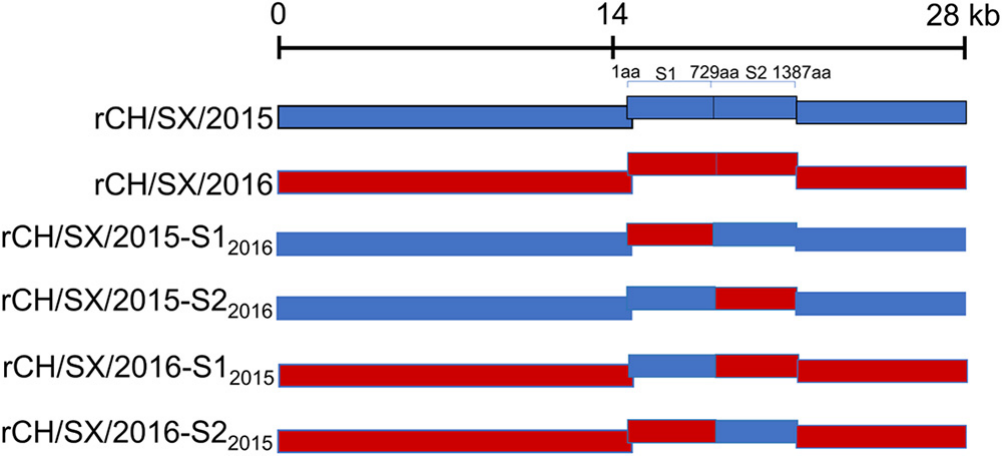
Schematic illustration of construction of rCH/SX/2015-S1_2016_, rCH/SX/2015-S2_2016_ and rCH/SX/2016-S1_2015_, rCH/SX/2016-S2_2015_. S1:1-729aa, S2:730-1387aa.

To further explore the key region that determines PEDV adaptation to cells, we constructed chimeric viruses that exchanged S1 and S2 subunits of rCH/SX/2015 and rCH/SX/2016, including rCH/SX/2015-S1_2016_, rCH/SX/2015-S2_2016_, rCH/SX/2016-S1_2015_ and rCH/SX/2016-S2_2015_ (Fig. 5). Unfortunately, none of the four chimeric strains were successfully recovered. However, as a positive control, the rescued rate of rCH/SX/2015 is 100% (Table 1). We then sequenced the recombinant plasmids to ensure that the failure to recover the chimeric strains was not due to sequence mutations (data not shown). These results showed that the cellular adaptability of PEDV depends on both S1 and S2.

**TABLE 1:** PEDV BAC recovery rates in Vero cells

BAC	No. of viruses recovered/no. of attempts	CPE	RT-PCR	Western blot	Recovery (%)
rCH/SX/2015	9/9	Yes	Yes	Yes	100
rCH/SX/2016	0/9	No	No	No	0
rCH/SX/2015-S1_2016_	0/8	No	No	No	0
rCH/SX/2015-S2_2016_	0/7	No	No	No	0
rCH/SX/2016-S1_2015_	0/7	No	No	No	0
rCH/SX/2016-S2_2015_	0/7	No	No	No	0

### The cellular adaptability of rCH/SX/2016-S_HNXP_ depends on S1 and the first half of S2 (S3).

**FIG 6:**
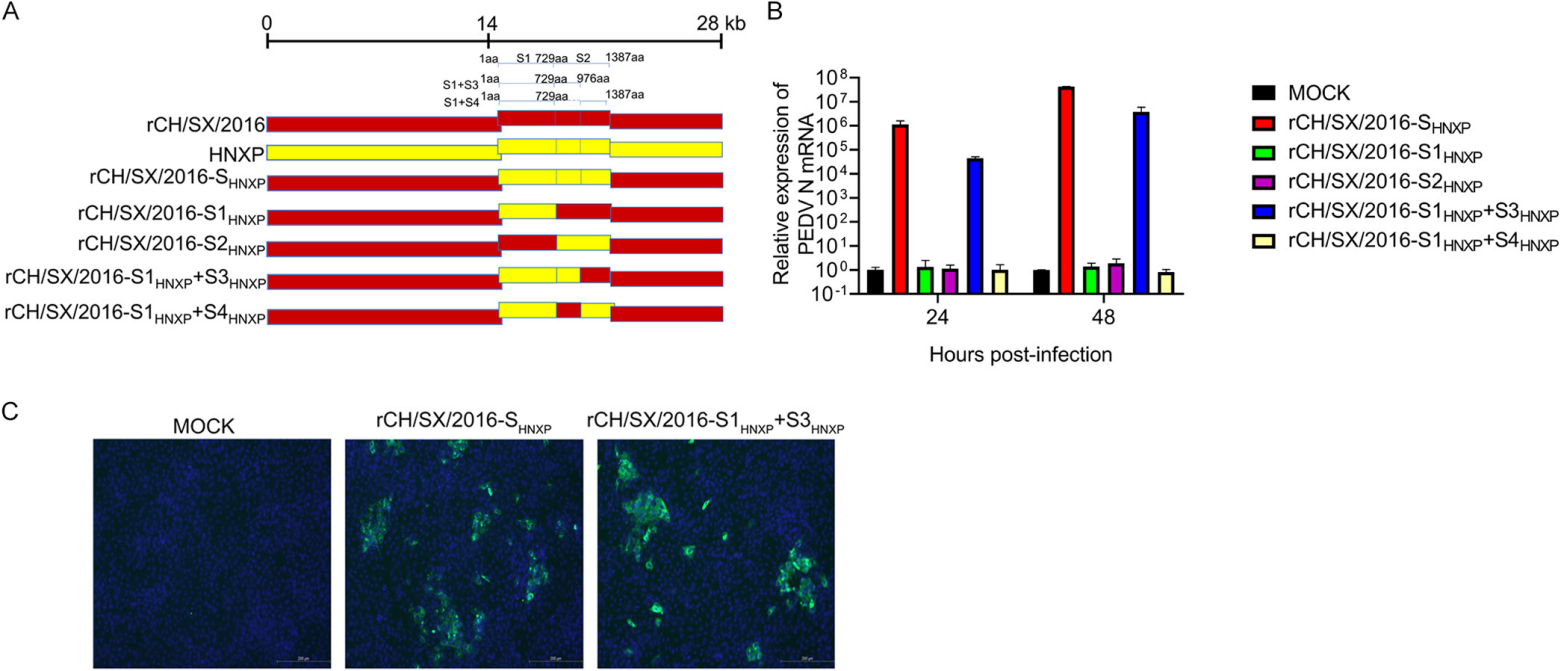
Adaptation of rCH/SX/2016-S1_HNXP_+S3_HNXP_ to Vero cells. (A) Schematic illustration of the construction of rCH/SX/2016-S_HNXP_, rCH/SX/2016-S1_HNXP_, rCH/SX/2016-S2_HNXP_, rCH/SX/2016-S1_HNXP_+S3_HNXP,_ and rCH/SX/2016-S1_HNXP_+S4_HNXP_. S:1-1387aa, S1:1-729aa, S2:730-1387aa, S1_HNXP_+S3_HNXP_:1-976aa, S1_HNXP_+S4_HNXP_:1-729aa and 977-1387aa. (B) Vero cells were infected with rCH/SX/2016-S_HNXP_, rCH/SX/2016-S1_HNXP_, rCH/SX/2016-S2_HNXP_, rCH/SX/2016-S1_HNXP_+S3_HNXP_, and rCH/SX/2016-S1_HNXP_+S4_HNXP_. Cells were harvested at 24 and 48 hpi. Then RT-qPCR was performed to detect the relative expression of mRNA of PEDV N protein (C). Vero cells were infected with rCH/SX/2016-S_HNXP_ and rCH/SX/2016-S1_HNXP_+S3_HNXP_. Infected cells were fixed at 36 h postinfection and immunolabeled with Fluorescein (FITC)-AffiniPure Goat Anti-Mouse IgG (H+L). Nuclei were labeled with DAPI (blue).

To further confirm the reliability of the above results, we construct chimeric infectious cDNA clones between the HNXP and the rCH/SX/2016 by replacing the S gene of rCH/SX/2016 with the HNXP S gene, and the resulted constructs were named rCH/SX/2016-S_HNXP_. rCH/SX/2016-S_HNXP_ was successfully rescued (Fig. 6B and C), which further verified the role of the S gene in PEDV adaptation to Vero cells. Next, we replace S1 and S2 of rCH/SX/2016 with S1 and S2 of HNXP. As expected, neither chimeric strain could be successfully rescued. We further constructed rCH/SX/2016-S1_HNXP_+S3_HNXP_ and rCH/SX/2016-S1_HNXP_+S4_HNXP_ chimeric strains (Fig. 6A). Surprisingly, it was the rCH/SX/2016-S1_HNXP_+S3_HNXP_ but not the rCH/SX/2016-S1_HNXP_+S4_HNXP_ that was successfully rescued (Fig. 6B) (Table 2). Besides, the reversion mutations experiment of rCH/SX/2016-S1_HNXP_+S3_HNXP_ was performed. In detail, the S3 region of HNXP was restored to the S3 region of rCH/SX/2016. The reversed strain rvCH/SX/2016-S1_HNXP_+S3_HNXP_ was not successfully rescued (Table 2). All the above results indicated that the cellular adaptability of rCH/SX/2016-S_HNXP_ depends on the cooperation between S1 and the first half of S2.

**TABLE 2:** PEDV BAC recovery rates in Vero cells

BAC	No. of viruses recovered/no. of attempts	CPE	RT-PCR	Western blot	Recovery (%)
rCH/SX/2016-S_HNXP_	8/8	Yes	Yes	Yes	100
rCH/SX/2016-S1_HNXP_	0/8	No	No	No	0
rCH/SX/2016-S2_HNXP_	0/8	No	No	No	0
rCH/SX/2016-S1_HNXP_+S3_HNXP_	7/7	Yes	Yes	Yes	100
rCH/SX/2016-S1_HNXP_+S4_HNXP_	0/8	No	No	No	0
rCH/SX/2016-S1_HNXP_+S3_P803L_	0/8	No	No	No	0
rCH/SX/2016-S1_HNXP_+S3_Y976H_	0/8	No	No	No	0
rCH/SX/2016-S1_HNXP_+S3_P803L+Y976H_	3/3	Yes	Yes	Yes	100
rvCH/SX/2016-S1_HNXP_+S3_HNXP_	0/3	No	No	No	0
rvCH/SX/2016-S1_HNXP_+S3_P803L_	0/3	No	No	No	0
rvCH/SX/2016-S1_HNXP_+S3_Y976H_	0/3	No	No	No	0

### Characterization of rCH/SX/2016-S1_HNXP_+S3_HNXP_ chimeric strain growth properties *in vitro*.

**FIG 7:**
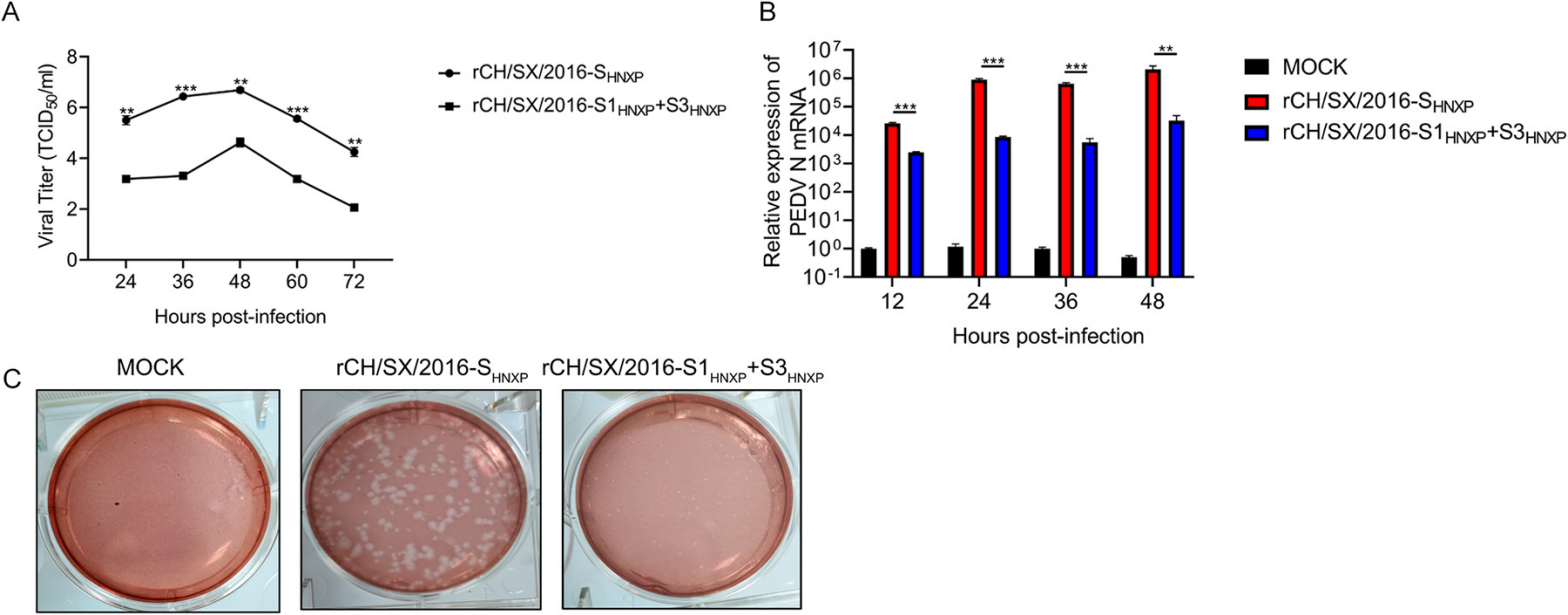
Growth characteristics of rCH/SX/2016-S1_HNXP_+S3_HNXP_. (A) Vero cells in 12-well plates were infected with rCH/SX/2016-S1_HNXP_+S3_HNXP_ and rCH/SX/2016-S_HNXP_. The supernatant was harvested at 24, 36, 48, 60, and 72 hpi and titrated on Vero cells. (B) MARC145 cells were infected with rCH/SX/2016-S_HNXP_ and rCH/SX/2016-S1_HNXP_+S3_HNXP_ at an MOI of 0.01. Cells were harvested at 12, 24, 36, and 48 hpi. Then RT-qPCR was performed to detect the relative expression of mRNA of PEDV N protein. (C) Representative images of the plaque morphologies of rCH/SX/2016-S_HNXP_ and rCH/SX/2016-S1_HNXP_+S3_HNXP_. Error bars indicate standard deviations. The level of significance was expressed as *, *P < *0.05, **, *P < *0.01 or ***, *P < *0.001.

As shown in fig. 7A and B, and 7C, compared with rCH/SX/2016-S_HNXP_, the replication of rCH/SX/2016-S1_HNXP_+S3_HNXP_ is significantly reduced, and its plaque sizes were significantly smaller, which means that the ability of rCH/SX/2016-S1_HNXP_+S3_HNXP_ to infect surrounding cells is significantly reduced.

### 803L and 976H are critical for rCH/SX/2016-S1_HNXP_+S3_HNXP_ adaptation to Vero cells.

**FIG 8:**
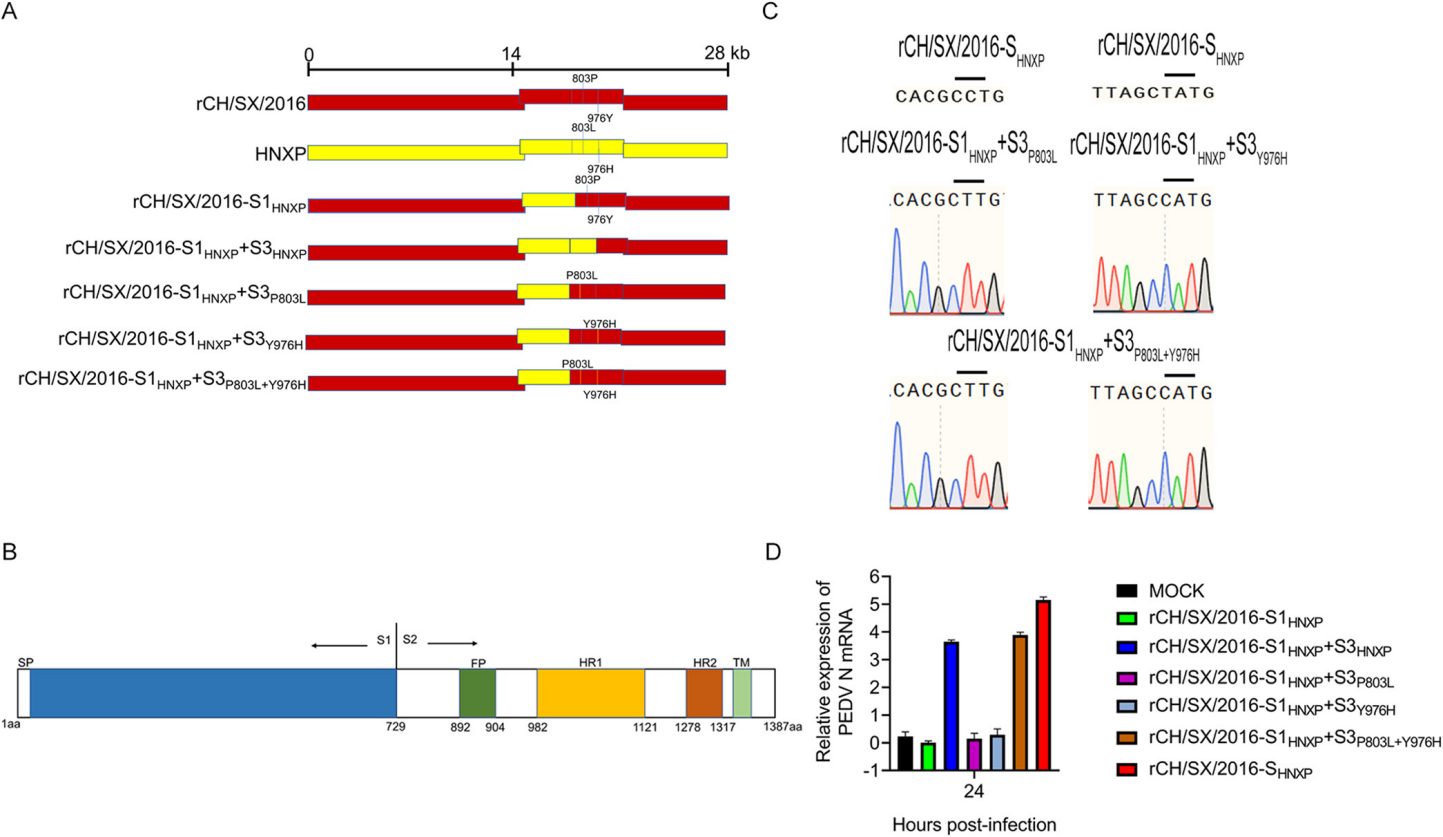
Adaptation of rCH/SX/2016-S1_HNXP_+S3_P803L_, rCH/SX/2016-S1_HNXP_+S3_Y976H_, and rCH/SX/2016-S1_HNXP_+S3_P803L+Y976H_ chimeric strains to Vero cells. (A) Schematic illustration of constructing rCH/SX/2016-S1_HNXP_+S3_P803L_, rCH/SX/2016-S1_HNXP_+S3_Y976H_ and rCH/SX/2016-S1_HNXP_+S3_P803L+Y976H_. (B) Schematic illustration of the structure of the PEDV S gene. SP: signal peptide, FP: the fusion peptide, HR1: heptad repeat 1, HR2: heptad repeat 2, TM: transmembrane domain. (C) Sequence analysis of rCH/SX/2016-S1_HNXP_+S3_P803L_, rCH/SX/2016-S1_HNXP_+S3_Y976H_, and rCH/SX/2016-S1_HNXP_+S3_P803L+Y976H_ plasmids. (D) Vero cells were infected with rCH/SX/2016-S_HNXP_, rCH/SX/2016-S1_HNXP_, rCH/SX/2016-S1_HNXP_+S3_HNXP_, rCH/SX/2016-S1_HNXP_+S3_P803L_, rCH/SX/2016-S1_HNXP_+S3_Y976H_, and rCH/SX/2016-S1_HNXP_+S3_P803L+Y976H_. Cells were harvested at 24 hpi. Then RT-qPCR was performed to detect the relative expression of mRNA of PEDV N protein.

To further determine which amino acid of S3 is critical for rCH/SX/2016-S1_HNXP_+S3_HNXP_ cellular adaptation, we compared the amino acids of S3 of HNXP and rCH/SX/2016 (data not shown). Only two amino acids differ in the S3 region of the two strains (Fig. 8A). Then, we constructed rCH/SX/2016-S1_HNXP_+S3_P803L_, rCH/SX/2016-S1_HNXP_+S3_Y976H_, and rCH/SX/2016-S1_HNXP_+S3_P803L+Y976H_ plasmids (Fig. 8A). Sequence analysis of rCH/SX/2016-S1_HNXP_+S3_P803L_, rCH/SX/2016-S1_HNXP_+S3_Y976H_, and rCH/SX/2016-S1_HNXP_+S3_P803L+Y976H_ plasmids indicated the mutation plasmids were successfully constructed (Fig. 8C). However, only rCH/SX/2016-S1_HNXP_+S3_P803L+Y976H_ strain can be successfully rescued (Fig. 8D) (Table 2). The reversion mutations experiments of rCH/SX/2016-S1_HNXP_+S3_P803L_ and rCH/SX/2016-S1_HNXP_+S3_Y976H_ were performed. In detail, the 803L of rCH/SX/2016-S1_HNXP_+S3_P803L_ was restored to 803P, and the 976H of rCH/SX/2016-S1_HNXP_+S3_Y976H_ was restored to 976Y. The reversed strains rvCH/SX/2016-S1_HNXP_+S3_P803L_ and rvCH/SX/2016-S1_HNXP_+S3_Y976H_ were not successfully rescued (Table 2), indicating that 803L and 976H are essential for rCH/SX/2016-S1_HNXP_+S3_HNXP_ adaptation to Vero cells.

## DISCUSSION

Cross-species transmission is a mechanism for coronavirus persistence and survival. Coronaviruses S protein plays a crucial role in coronaviruses' cell and tissue tropism, pathogenesis, and cross-species transmission (24, 25). ACE2s from various species could bind to the SARS-CoV-2 receptor-binding domain (RBD) and facilitate the transduction of SARS-CoV-2(6). The N501Y spike substitution imparts cross-species transmission of SARS-CoV-2 to mice (26), strengthens its binding to receptor ACE2 (27), and enhances SARS-CoV-2 infection and transmission (28). The cells of bovine (29), human, monkey, avian or canine origin permitted PDCoV infection (1). Consistent with the results at the cellular level, calves (29) and Haitian children (5) are observed to be infected with PDCoV *in vivo*. Similar to PDCoV, PEDV is also replicated in cell lines from different animal species, such as bats, humans, monkeys, and birds (10, 11). There is the potential for evolutionary change and adaptation of PEDV, leading to human and other animal infections. In this study, we found the key gene of PEDV to adapt to cells, which provides a reference for the study of PEDV host tropism.

Although PEDV infected many species' cells, *in vitro* isolation of PEDV remains a huge challenge. In this study, several attempts were made to isolate the PEDV CH/SX/2016 virus from the small intestine homogenates and rescue rCH/SX/2016 by adding trypsin at different concentrations in Vero and LLC-PK1 cells but failed. Considering the function of coronavirus S protein, we speculated that S protein might play a key role in viral adaptation to cells. Reverse genetics is a valuable tool to identify viruses' virulence genes and generate vaccine candidates (30–33). Using reverse genetic manipulation techniques, we confirmed that the S gene determines the adaptability of PEDV to monkey Vero cells by exchanging the S genes of rCH/SX/2015 strain adapted to Vero cells and rCH/SX/2016 strain unadapted to Vero cells (Fig. 3B and C). Interestingly, the titer of the rCH/SX/2016-S_2015_ strain was lower than that of rCH/SX/2015 (Fig. 3D), suggesting that other genes of the CH/SX/2016 strain may involve in viral replication, such as the ORF3 gene since CH/SX/2015 strain has a 49 bp deletion of ORF3 gene compared with CH/SX/2016 strain, this deletion may affect viral replication (34).

Currently, PEDV has been reported to mainly infect porcine cells such as IEC(11), IPEC-J2 (35, 36), and LLC-PK1 (37). However, we found that the infection of PEDV to LLC-PK1 cells was strain-specific. LLC-PK1 cells are susceptible to HNXP strain but not CH/SX/2015 strain. Then, we study the role of the PEDV S gene on the adaptability of porcine cells. Compared with the parent rCH/SX/2015, the chimeric strain rCH/SX/2015-S_HNXP_ showed infectivity to LLC-PK1, but the infection rate was lower than that of the HNXP strain (Fig. 4F). It is possible that other genes of the virus also played a role in the infection of LLC-PK1 cells.

All studies on cell tropism of coronaviruses were based on strains that have adapted to cells. The S2 subunit of infectious bronchitis virus (IBV) Beaudette is identified as a determinant of cellular tropism to Vero cells (38). SARS-CoV-2 D614G variant exhibits more efficient infection, replication, and competitive fitness in primary airway epithelial cells (39). To explore the key regions or amino acid sites that determine PEDV from unadapted to adaptive cells, we generated chimeric viruses that exchanged S1 and S2 subunits of rCH/SX/2015 and rCH/SX/2016 (Fig. 5). Surprisingly, none of these chimeric strains has been successfully rescued, indicating that the adaptability of PEDV to cells requires both S1 and S2 (Table 1).

**FIG 9:**
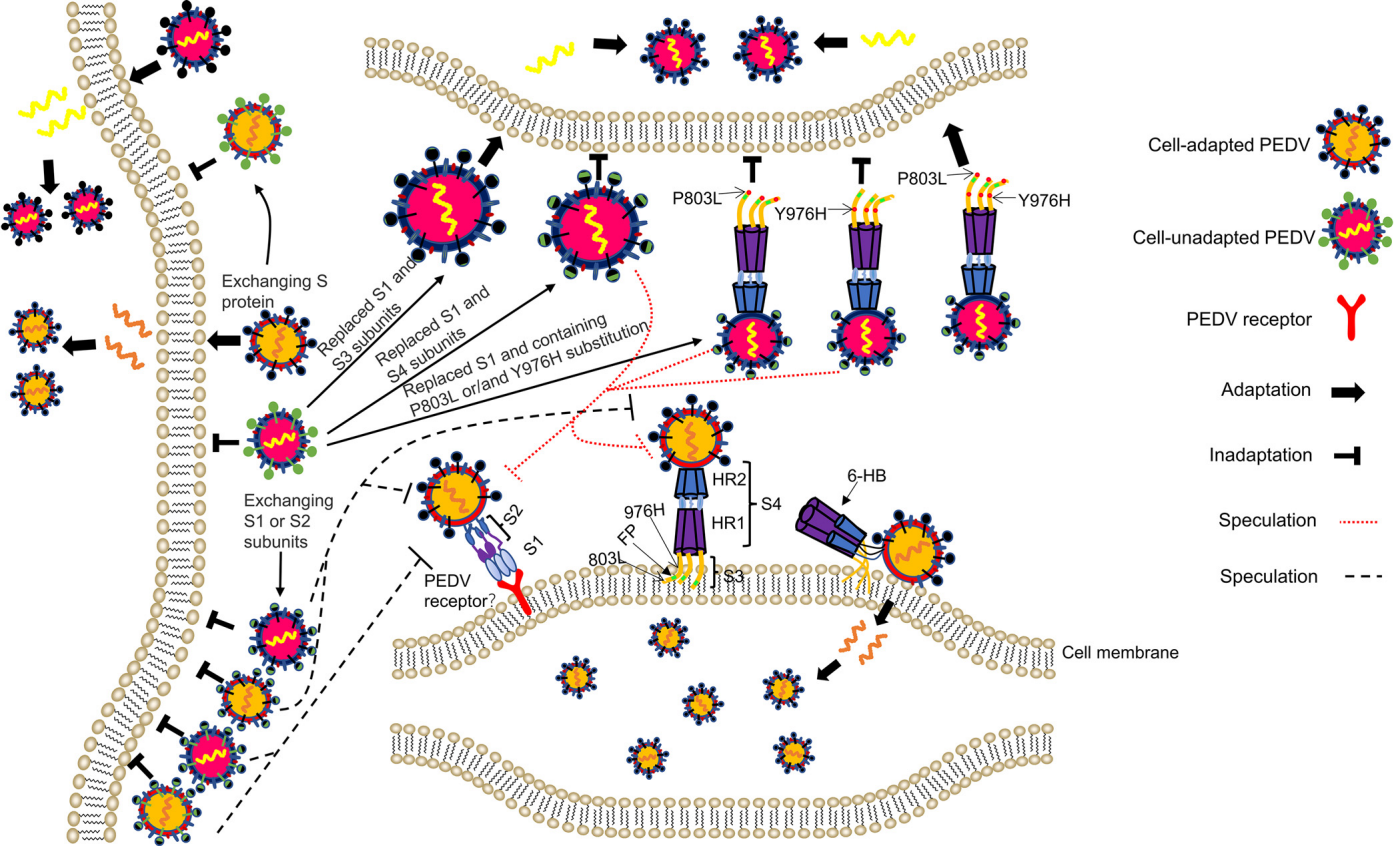
Schematic illustration of the molecular mechanism of PEDV cell tropism. The S genes of cell-adapted PEDV strains impart the cellular adaptation of cell-unadapted PEDV strains. The S genes of cell-unadapted PEDV strains abolish the cellular adaptation of cell-adapted PEDV strains. The cellular adaptability of PEDV depends on the cooperation between S1 and S2 (or the first half of S2). We speculate that the first half of S2 (S3) might involve the binding of S1 to receptors or in membrane adsorption mediated by the FP, in the process of which the 803L and 976H perhaps played an important role. FP: the fusion peptide, HR1: heptad repeat 1, HR2: heptad repeat 2, 6-HB: six-helix bundle.

According to existing literature reports, we generally believe that the process of coronaviruses invading cells includes the following processes. First, the S1 subunit binds the cellular receptor, followed by conformational changes in the S2 subunit, which allows the fusion peptide to insert into the host target cell membrane. The HR1 region forms a homotrimeric assembly with the HR2. Then, this six-helix bundle (6-HB) core structure helps bring the viral and cellular membranes into proximity for viral fusion and entry (Fig. 9) (40, 41). Peptides derived from the HR1 and HR2 domains exhibited inhibitory activity against multiple human coronaviruses (41, 42), highlighting that the HR1 and HR2 domains play a pivotal role in coronaviruses infections. From the above statement, we know that the cooperation between S1 and S2 or HR1 and HR2 is crucial to the adaptation of coronavirus. The S4 of PEDV contains the heptad repeat 1 (HR1) region and heptad repeat 2 (HR2) (Fig. 8B). Surprisingly, rCH/SX/2016-S1_HNXP_ and rCH/SX/2016-S1_HNXP_+S4_HNXP_ were not rescued (Fig. 6) (Table 2). Alignment amino acid sequence of S4 region of rCH/SX/2016-S_HNXP_ and rCH/SX/2016, the HR1 and HR2 regions have the same amino acid sequence but differ in only a few amino acids in the remaining region, which demonstrate that the cooperation between S1 and HR regions and the different amino acids did not play a pivotal role in rCH/SX/2016-S_HNXP_ cell tropism. The ability of rCH/SX/2016-S1_HNXP_+S3_HNXP_ to infect surrounding cells is significantly reduced (Fig. 7C), suggesting that the different amino acids of S4 are involved in virus replication and transmission, although it does not play a decisive role in rCH/SX/2016-S_HNXP_ cell adaptation. Based on preserving the immunogenicity of S protein to the maximum extent, the rCH/SX/2016-S1_HNXP_+S3_HNXP_ may have the potential to be a live attenuated vaccine candidate.

Further study indicated that the cellular adaptability of rCH/SX/2016-S_HNXP_ depends on the S1 and the first half of S2 and the 803L and 976H of the S2 subunit are critical for rCH/SX/2016-S1_HNXP_+S3_HNXP_ adaptation to Vero cells (Fig. 6 and 8) (Table 2). The fusion peptides (FPs) participate in the adsorption of the virus to the cell membrane (Fig. 9) and show a strong homology across different coronaviruses (43). We initially speculated that the cooperation between S1 and FP in the first half of S2 might play an important role in rCH/SX/2016-S_HNXP_ and rCH/SX/2016-S1_HNXP_+S3_HNXP_ cell tropism. However, rCH/SX/2016-S_HNXP_ and rCH/SX/2016-S1_HNXP_+S3_HNXP_ shares the same FP amino acid sequence to rCH/SX/2016-S1_HNXP_ and rCH/SX/2016-S1_HNXP_+S4_HNXP_ and 803L and 976H were not located in the fusion peptide, which showed the complexity of PEDV cell adaptation.

Receptor interaction plays a key role in coronaviruses' cell, tissue, and host tropism. Unfortunately, even though porcine aminopeptidase N (pAPN), transmembrane protein 41B (TMEM41B), transmembrane serine protease (TMPRSS2), a trypsin-like serine protease (MSPL), and transferrin receptor I can effectively facilitate PEDV invasion of cells (22, 44, 45) and sialic acid-binding activity of the PEDV S1 subunit facilitates cell entry (46), the functional receptors of PEDV are still unclear. Isolated exchange of S1 and S2 of adaptive strains did not make PEDV adapt to cells (Fig. 5 and 6) (Table 1 and 2). The reason why rCH/SX/2015-S_2016_, rCH/SX/2015-S1_2016_, and rCH/SX/2016-S2_2015_ was not adapted to Vero cells probably because that the rCH/SX/2016 S1 subunit did not bind to the receptor (Fig. 9). Besides, rCH/SX/2016-S_HNXP_ and rCH/SX/2016-S1_HNXP_+S3_HNXP_ share the same S1 subunit and HR regions with rCH/SX/2016-S1_HNXP_, rCH/SX/2016-S1_HNXP_+S4_HNXP_, rCH/SX/2016-S1_HNXP_+S3_P803L_, and rCH/SX/2016-S1_HNXP_+S3_Y976H_. Assuming that these strains can normally form 6-HB, we hypothesize that the first half of S2 (S3) might involve in the binding of S1 to receptors or in membrane adsorption mediated by FP, in the process of which the 803L and 976H perhaps played an important role (Fig. 9), suggesting that S1 and S2 may not be separated when identifying PEDV receptors.

In conclusion, we demonstrated that PEDV cell tropism depends on the S protein and the interaction between S1 and S2 by constructing a series of chimeric recombinant viruses via exchanging the S, S1, or S2 genes of cells-adapted PEDV strains and cells-unadapted PEDV strains. Furthermore, we have shown that 803L and 976H are essential for rCH/SX/2016-S1_HNXP_+S3_HNXP_ adaptation to Vero cells. Our finding might provide insight into the potential role of coronaviruses S protein in cross-species transmissibility, identifying PEDV functional receptors, and developing vaccines of PEDV and other coronaviruses.

## MATERIALS AND METHODS

### Cells and viruses.

Vero (CCL-81), LLC-PK1, and MARC145 cells (47, 48) were preserved in our laboratory and cultured in Dulbecco's modified Eagle medium (DMEM; Gibco, USA) supplemented with 10% fetal bovine serum (FBS; TransGen Biotech, China), 100 U/mL penicillin, and 100 μg/mL streptomycin. PEDV strain CH/SX/2015 (GenBank No.MT783684) was adapted to Vero cells but not to LLC-PK1 cells; CH/SX/2016 strain (GenBank No.MT787025) was not successfully isolated *in vitro*; that is, the virus did not infect cells. PEDV HNXP strain was adapted to Vero cells and LLC-PK1 cells kindly provided by Dr. Changxu Song, College of Animal Science, South China Agriculture University, Guangzhou, China.

### Virus isolation.

Vero cells were grown to 90% confluence in 6-well plates and then were inoculated with 500 μL PEDV positive intestinal samples filtered through a 0.22 μm syringe filter (Millipore, USA). Then 1.5 mL DMEM without fetal bovine serum supplemented with 10 μg/mL trypsin was added per well. After adsorption for 60 min at 37°C, the cells were washed three times with PBS and then added 2 mL DMEM with 10 μg/mL trypsin. The CPE was observed, and the cells were harvested until the CPE exceeded 60% or no CPE was observed at 7 days postinoculation. The plates were subjected to freeze-thaw once, and then the supernatants were collected (passage 0 [P0]) for further propagation or stored at −80°C. If CPE and RT-PCR were negative after 4 passages, the virus isolation was considered a failure.

### Strategies for constructing chimeric full-length cDNA clones of PEDV.

The accurate, complete gene sequence of the CH/SX/2015 and CH/SX/2016 strain was obtained by RT-PCR and 5′-and3’-RACE. The full-length rCH/SX/2015 was divided into nine continuous fragments (A: nucleotide [nt]1–41, B: nt 42–4778, C: nt 4779–9500, D: nt 9501–13964, E: nt 13965–16866, F: nt 16867–21004, G: nt 21005–24772, H: nt 24773–27084, and I: nt 27085–27972). The bacterial artificial chromosome (BAC) provides an invaluable tool for genetic manipulation (49). All nine continuous fragments were subsequently cloned into the pBeloBAC11 vector. The final plasmid was named pBAC-CH/SX/2015. A similar strategy was used to generate other full-length cDNA clones.

### Transfection and rescue of recombinant viruses.

The recombinant BAC plasmids were prepared using the QIAfilter Plasmid Kits and verified different restriction endonucleases. Vero cells were grown to 90% confluence in a six-well plate, and 2.5 μg of the recombinant BAC plasmids were transfected into Vero cells with Lipofectamine 3000 transfection reagent by following the manufacturer's protocol to rescue infectious PEDV. The CPE was monitored daily after transfection. After 5 days, the cells and supernatants were collected and freeze-thawed three times, and the supernatants were then used to infect fresh Vero cells to propagate the rescued virus. The rescued virus was used for further analysis after three successive propagation generations.

### Identification of the marker mutation.

The viral RNA of three-passage (P3) virus stock was extracted using TRIzol reagent (TaKaRa, Japan) and reverse transcribed using a Primescript RT reagent kit (TaKaRa, Japan) according to the manufacturer's instructions. Two fragments were amplified by PCR to identify the presence of the markers in fragment C of rCH/SX/2015 (nucleotide positions 6786 or 8025), using primers CH/SX/2015-6786-F: ATAGGTAGCCCTGTTTGTGATG; CH/SX/2015-6786-R: GCTATCTGTTATGTCAAAGTTG; CH/SX/2015-8025-F: AGGAAGTACATTATTCGTACGA; CH/SX/2015-8025-R: CAACACGGCAATAAGTCATAG. Two fragments were amplified by PCR to identify the presence of two mutations in fragment C of rCH/SX/2016 (nucleotide positions 3493 or 7615), using primers: CH/SX/2016-3493-F: ACCGATCCTAATCTGCCCGTA; CH/SX/2016-3493-R: AGATGCCGGTGCCAACAATACT; CH/SX/2016-7615-F: ACAGATAGCAAATACACTTGC; CH/SX/2016-7615-R: ACTCTCAATATACTTGAAGT. The fragments were gel-purified and sequenced by one of the amplified primers.

### Indirect immunofluorescence assay.

Vero or LLC-PK1 cells were grown on coverslips in a 24-well plate and were infected with 0.1MOIPEDV or mock-infected for 36 h. Cells were washed with PBS and fixed with 4% paraformaldehyde (PFA) for 10 min at 37°C, then membrane permeabilization with 0.25% Triton X-100 in PBS for 10 min at 37°C. Cells were blocked with 1%BSA at 37°C for 30 min and then incubated with mouse anti-N polyclonal antibody at a dilution of 1:1000 at 37°C for 1 h. Cells were washed with PBS three times, followed by incubation with Rhodamine (TRITC) AffiniPure Goat Anti-Mouse IgG (H+L) or Fluorescein (FITC)-AffiniPure Goat Anti-Mouse IgG (H+L) at 1:200 at 37°C for 1 h. Cells were washed with PBS three times and stained with 4–6-diamidino-2-phenylindole (DAPI) for 5 min at room temperature. The images were taken by a Leica confocal microscope.

### Western blot.

Vero cells were grown to 90% confluence in 6-well plates and infected with PEDV at an MOI of 0.1. Cells were harvested when CPE reached 60%. The cells were lysed with 100 μL of ice-cold RIPA buffer for 30 min on ice, and then the supernatant proteins were collected after 12,000 × *g* for 10 min at 4°C. The supernatant proteins were separated by 12% SDS-PAGE and transferred onto a polyvinylidene difluoride (PVDF) membrane (Millipore, USA). The membrane was blocked with 5% skim milk at 25°C for 1 h and then incubated with mouse anti-N polyclonal antibody at a dilution of 1:2500 and anti-actin antibody at a dilution of 1:2500 in PBS containing 0.1% Tween 20 (PBST) at 25°C for 1 h. After washing with PBST, the membranes were further incubated with the secondary antibody HRP-conjugated goat anti-mouse IgG (Sigma-Aldrich, St. Louis, MO, USA) at a dilution of 1:5000 at 25°C for 1 h. According to the manufacturer's instructions, the immune-stained proteins were visualized using an ECL chemiluminescent detection system (Pierce, Rockford, IL, USA).

### Real-time RT-PCR assay.

Vero cells or MARC145 cells (50) were seeded in 24-well plates. The cells were washed with PBS and lysed in RNAiso Plus (TaKaRa, Japan), and reverse transcription was performed using HiScript II Q RT SuperMix for qPCR (Vazyme, China). Amplification was carried out in a 10 μL reaction mixture containing 5 μL ChamQ SYBR qPCR Master Mix (Vazyme, China), 0.2 μM concentration of each primer PEDV N-F: TGGTGGCTGCTGTCAAGG, PEDV N-R: TTTTCGACAAATTCCGCAT; Actin-F: ATCGTGCGTGACATTAAG, Actin-R: ATTGCCAATGGTGATGAC (51) and 2.5 μL cDNA. The reaction procedure was 95°C for 30 s, followed by 40 cycles at 95°C for 10 s, 60°C for 30 s. The relative mRNA expression level was normalized to the housekeeping gene β-actin. The relative transcript levels were analyzed using the ΔΔ Ct method.

### Growth kinetics.

Vero cells monolayers in 12-well plates were infected with each virus at a multiplicity of infection (MOI) of 0.1 to analyze the growth curves of PEDV. After 1 h absorption, the cell monolayers were washed with PBS three times and then added to the maintenance medium containing 10 μg/mL of trypsin. The supernatants were collected at the indicated time points, and the virus titers were determined by TCID_50_ assay. Briefly, Vero cells were plated on 96-well plates at 90% confluence and washed three times with PBS. Virus supernatants were 10-fold serially diluted, and 100 μL of each was added to each well with eight replicates. The number of wells with visible CPE was counted 3∼5 days, and virus titers were calculated.

### Plaque assay.

Vero cells monolayers in 6-well plates were infected with 2 mL of a 10-fold serially diluted parent or rescued PEDV viruses. After 1 h absorption, the cell monolayers were washed with PBS three times and then overlaid with 1% low melting agarose in DMEM supplemented 10 μg/mL of trypsin. At 5 dpi, plaques were visualized by the neutral red dye.

### Statistical analysis.

Statistical analyses were performed using GraphPad Prism 6.0. Differences among groups were examined using Student's *t* test or a one-way analysis of variance (ANOVA). Error bars indicate standard deviations. The level of significance was expressed as *, *P < *0.05, **, *P < *0.01 or ***, *P < *0.001.
